# Supplementary material for: Unfolding Protein-Based Hapten Coupling via Thiol–Maleimide Click Chemistry: Enhanced Immunogenicity in Anti-Nicotine Vaccines Based on a Novel Conjugation Method and MPL/QS-21 Adjuvants
Source: Polymers (Basel). 2024 Mar 28;16(7):931. doi: 10.3390/polym16070931 (PMC11013800; doi:10.3390/polym16070931)
Supplement: Supplementary file 1 [file polymers-16-00931-s001.zip › polymers-2902831-supplementary.pdf]

Unfolding Protein-Based Hapten Coupling via Thiol–Maleimide Click Chemistry: Enhanced Immunogenicity in Anti-Nicotine Vaccines Based on a Novel Conjugation Method and MPL/QS-21 Adjuvants

Ying Xu, Huiting Li, Zhen Wang, Xiongyan Meng, Tiantian Sun,Shuai Meng* and Chengli Zong *

Experimental Section

1. Chemistry

1.1. General considerations

Unless otherwise noted, all materials and solvents were used as received from Adamas-beta® without further purification. NMR spectra were recorded on Varian Mercury 500-MHz spectrometers. Chemical shifts are reported in parts per million (ppm) relative to MeOH-d_4, $CDCl_3$, or TMS signature chemical shifts. NMR data are presented as follows: chemical shift, multiplicity (s = singlet, d = doublet, t = triplet, dd = doublet of doublet, m = multiplet and/or multiple resonances), coupling constant in hertz (Hz), integration. ^{13}C data were derived from the HSQC spectrum. All NMR signals were assigned on the basis of ^{1}H NMR, COSY, and HSQC experiments. TLC-analysis was performed on silica gel 60 F254 (Huang Hai Inc.) with detection by UV absorption (254 nm) when applicable, and by spraying with a solution of $(NH_4)_6Mo_7O_{24}\cdot H_2O$(25 g / L) in 5% sulfuric acid in ethanol followed by charring. All reactions were carried out under an argon atmosphere.

1.2. Procedures

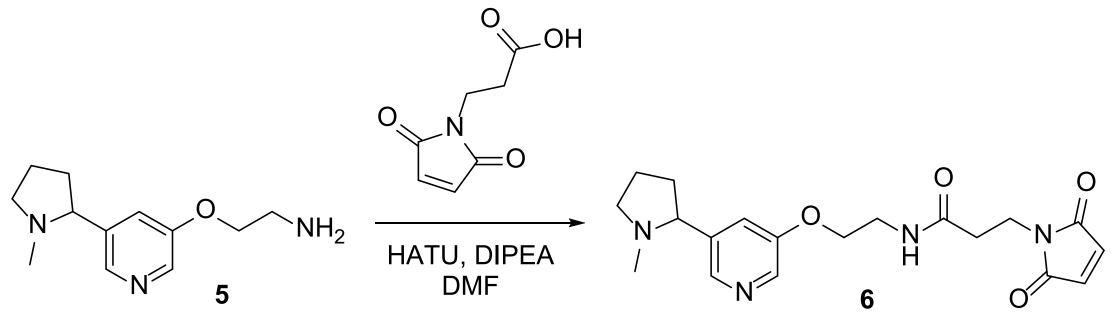

2-[(5-(1-methylpyrrolidin-2-yl)pyridin-3-yl)oxy]ethan-1-amine (1)

Compound **1** was synthesized according to the literature.[1] The NMR spectra matched the reported data.

3-(2,5-dioxocyclopent-3-en-1-yl)-*N*-(2-((5-(1-methylpyrrolidin-2-yl)pyridin-3-yl)oxy)ethyl)propanamide (2)

Maleimidopropionic acid (1.74 g, 1.5 equiv.) and HATU (5.22 g, 2.0 equiv.) were dissolved in anhydrous DMF (80 mL). The mixture was stirred for 10 minutes at room temperature followed by the dropwise addition of compound **1** (1.5 g, 1.0 equiv.) and DIPEA (598 µL, 0.5 equiv.). The resulting mixture was stirred until TLC (DCM / MeOH, 10/1, *v*/*v*) indicated the disappearance of **1** (~6h). The mixture was concentrated under reduced pressure, and the residue was purified by silica gel column chromatography using a gradient of CH_2Cl_2 and MeOH (from 50/1 to 1/1, *v*/*v*) to afford **2** (1.25 g, 49.8%).

^{1}H NMR (500 MHz, MeOD) δ 8.34 (s, 1H), 8.29 (s, 1H), 7.67 (s, 1H), 6.72 (s, 2H), 4.39 (t, J = 8.9 Hz, 1H), 4.14 (m, 2H), 3.83 (m, 1H), 3.75 (t, J = 6.8 Hz, 2H), 3.55 (t, J = 5.5 Hz, 2H), 3.25 (m, 1H), 2.75 (s, 3H), 2.56 (m, 1H), 2.47 (t, J = 6.7 Hz, 2H), 2.34 (m,1H), 2.34-2.23 (m, 2H). ^{13}C NMR (125 MHz, MeOD) δ 173.4, 172.1, 157.1, 142.7, 140.8, 135.4, 132.0, 122.1, 70.9, 68.2, 57.3, 39.6, 39.1, 35.8, 35.5, 32.2, 22.7. ESI calculated for $[M+K]^+$: 411.1, found: 411.3

2. NMR spectra

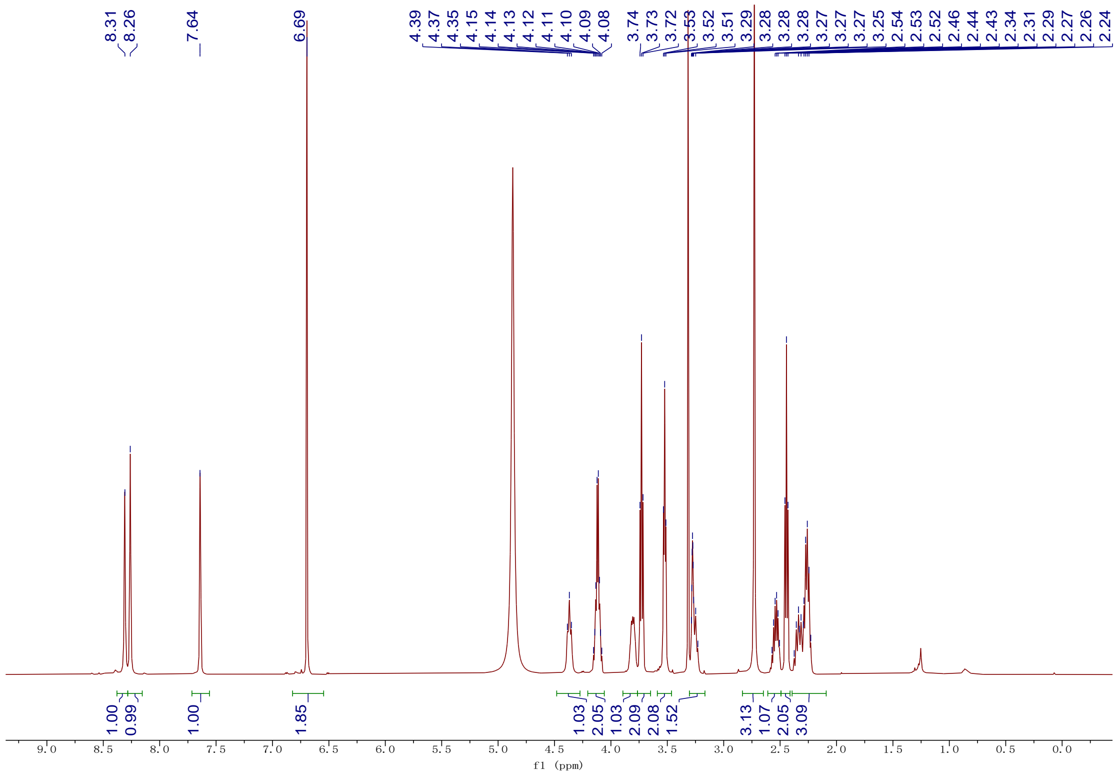

^{1}H NMR of compound **2** (CD3OD, 500 MHz)

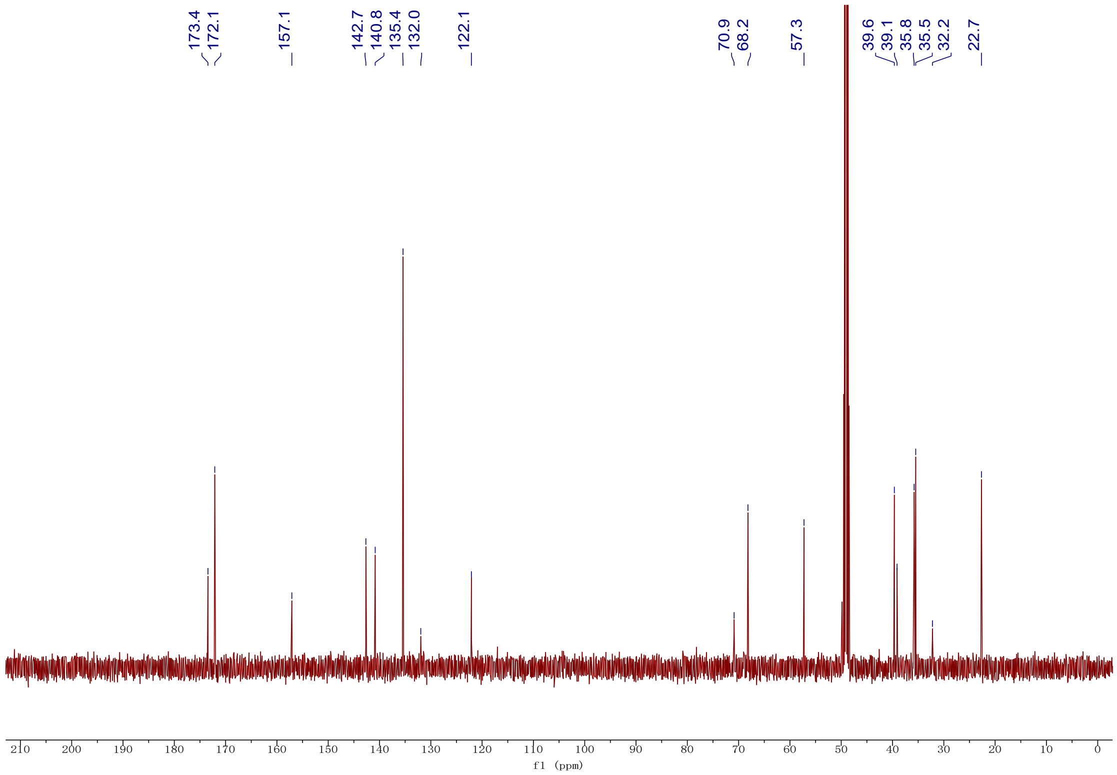

^{13}C NMR of compound **2** (CD3OD, 500 MHz)

References

1. Pryde, D.C.; Jones, L.H.; Gervais, D.P.; Stead, D.R.; Blakemore, D.C.; Selby, M.D.; Brown, A.D.; Coe, J.W.; Badland, M.; Beal, D.M.; Glen, R.; Wharton, Y.; Miller, G.J.; White, P.; Zhang, N.; Benoit, M.; Robertson, K.; Merson, J.R.; Davis, H.L.; McCluskie, M.J. Selection of a Novel Anti-Nicotine Vaccine: Influence of Antigen Design on Antibody Function in Mice. *PLoS One* **2013**, *8 (10)*, e76557.
